# A disorder of anger and aggression: Children’s perspectives on attention deficit/hyperactivity disorder in the UK

**DOI:** 10.1016/j.socscimed.2011.03.049

**Published:** 2011-09

**Authors:** Ilina Singh

**Affiliations:** London School of Economics and Political Science, BIOS Research Centre, London, UK

**Keywords:** United Kingdom, Attention deficit/hyperactivity disorder, ADHD, Diagnosis, Stigma, Aggression, Bullying, Mental capital, Children

## Abstract

This article investigates the social and moral dimensions of Attention Deficit/Hyperactivity Disorder (ADHD) diagnosis, asking what ADHD means in UK children’s everyday lives, and what children do with this diagnosis. Drawing on interviews with over 150 children, the analysis examines the influence of a UK state school-based culture of aggression on the form and intensity of diagnosed children’s difficulties with behavioral self-control. Diagnosed children’s mobilization of ADHD behaviors and their exploitation of the diagnosis shows how children’s active moral agency can support and compromise cognitive, behavioral and social resilience. The findings support a proposal for a complex sociological model of ADHD diagnosis and demonstrate the relevance of this model for national policy initiatives related to mental health and wellbeing in children.

## Highlights

► In the UK, ADHD is understood to be a disorder of anger and aggression. ► A UK state school-based culture of aggression intensifies children’s difficulty controlling their behavior. ► Children use ADHD diagnosis and symptomatic behaviors to social and moral ends. ► Children rely on friendships to help manage their difficulty with self-control. ► Mental health policy should address the social and moral, as well as the cognitive aspects of ADHD diagnosis.

## Introduction

Attention Deficit/Hyperactivity Disorder (ADHD) has been a ‘hot’ sociological and ethical topic now for over a decade. The ambiguity of core symptoms – inattention, hyperactivity and impulsiveness – the international inconsistency of diagnostic processes and guidelines, and the growing global use of psychotropic drugs to treat ADHD mean that this disorder remains one of the world’s most debated childhood psychiatric diagnoses. A controversial but authoritative meta-analysis of ADHD prevalence rates by geographic region estimates that the world-wide prevalence of ADHD is approximately 5% of school age children. By this estimate ADHD is the most common child psychiatric disorder in the world (Polancyck et al., 2007).

In the increasingly intersecting literatures of sociology and bioethics, the debate over ADHD has covered a range of themes, including medicalization (Conrad, 1976), neurochemistry and identity (Rose, 2007), the DSM (Kirk and Kutchins 2003), big pharma (Healy, 2002), and neuroenhancement (President’s Council Report, 2003). This is a diverse but distinguished literature, in which ADHD generally serves as a case study illustrating potential social and ethical consequences of psychiatric diagnosis and treatments; or macro-level analyses of corporate, governmental and institutional interactions that inhere in the take up of psychiatric diagnosis and drug treatments.

These analytic approaches make substantial contributions in their own right. However, they frequently misrepresent the phenomenological ground of ADHD, and bracket discussion of its biological dimensions. Although there have been important efforts to deal theoretically with the biological dimensions of complex human behaviors in sociology (eg Hacking, 1999, Horwitz, 2002, Rose, 2007), neither bioethics nor sociology has yet managed to fully take on the complexity of ADHD that is now widely accepted in the world of developmental and clinical child psychiatry (Singh, 2008). Here, ADHD is a ‘complex heterogeneous disorder’ characterized by different gene-environment-gene pathways (eg Sonuga-Barke, 2005). But in sociology and bioethics, ADHD is still surrounded by a discourse of suspicion fueled by worries about social construction and medicalization.

Recent health economic data indicates that ADHD diagnoses are increasing rapidly around the globe, a phenomenon which offers rich opportunities to diversify and to localize analyses of ADHD diagnosis (Scheffler, Hinshaw, Modrek, & Levine, 2007). But international sociological perspectives on ADHD offer little relief from the discourse of suspicion; indeed, in the few published analyses of ADHD in non-US contexts, there is much analysis of medicalization, biopower, and the role of schooling in fostering medicalization (eg Graham, 2008, Johannesson, 2006, Maturo, 2009, Timimi, 2009, Vega Balbas, 2007). There is little attention to ADHD as a lived experience in local contexts, or consideration of how interactions between individual biology and particular environmental inputs might give shape and meaning to symptomatic behaviors, and to the success of interventions. This is a significant gap in the literature on ADHD especially, given that children carry this diagnosis in the midst of complex and highly contested social, political and medical territories. The phenomenology of diagnosis is likely to contribute important insights to some very basic concerns about the consequences of diagnosis for children’s overall wellbeing.

As Margaret Lock’s seminal work has shown, analyses of ‘local biology’ (Lock, 2001) problematize assumptions about universal, natural health phenomena without falling back into dichotomous nature-nurture arguments. Understanding the dialectic between features of particular contexts and biological dispositions also has significant policy implications, in the sense that knowing more about how this dialectic contributes to behavioral outcomes can help guide interventions to promote children’s overall functioning and wellbeing in particular social and cultural contexts. A further value of attention to this dialectic is that is opens up the possibility of children’s agency and resilience in the face of state and institutional practices. Most of the sociological and bioethics literature on ADHD diagnosis paradoxically silences children, even while arguing for their liberty, because children’s experiences and voices hardly ever enter the frame. From this position, children are made ‘docile bodies’ (Foucault, 1975) ripe for both intellectual and clinical manipulation.

This docility has also meant that analysis of the moral dimensions of diagnosis – which have been articulated in the sociological literature through important concepts like labeling theory, stigma and analyses of agency (eg Goffman, 1961, Scheff, 1974) - has tended not to integrate children’s lived experiences of ADHD. Indeed analyses of the moral subjectivities of diagnosed children have to a great extent bracketed the possibility of children’s active negotiation of stigma, labeling and agency. Less surprisingly, the bioethics literature on the moral dimensions of ADHD diagnosis has similarly avoided discussion of children’s lived experiences, drawing instead on autonomy arguments to articulate the dangers of learning behavioral self-control via a diagnosis that reduces self-control to biology and offers a pill to force children into obedience with social norms (Fukuyama, 2002).

As I have argued elsewhere, analyses of the moral dimensions of ADHD diagnosis that include no understanding of how children live with ADHD are inevitably short-sighted (Singh, 2005). As a growing number of ethicists and sociologists argue, morality is “essentially social” (Lindemann, 2010), and “our capacities for moral judgment do not track truths in the world independent of us…” (Walker, 2009:2). The development of moral subjectivity “emerge(s) in the context of relations to others” (Butler, 2005:20). Self-control, which is the key behavioral node in ADHD diagnosis (Barkley, 1997), is arguably also a central mechanism of moral capacity, and essential to moral agency. If we theorize the capacity for self-control to emerge out of a negotiation between biological dispositions and social factors, then there can be no good understanding of the moral dimensions of ADHD diagnosis without empirical research among children.

The analysis in this article is informed by an empirical bioethics approach, which integrates the empirical and theoretical tools of social science with bioethical concerns (Haimes, 2002) in order to deepen and to specify analyses of the social and ethical impacts of biomedical styles of thought and biomedical technologies in context. Two major questions organize the analytic sections in this article: What do ADHD diagnosis and symptomatic behaviors *mean* in the spaces and relationships that make up children’s everyday lives; and, what do children *do* with an ADHD diagnosis, and how do the spaces they inhabit – physical spaces, social spaces and national spaces – help to create and to constrain those possibilities? I am specifically interested in uncovering the social and moral dimensions of ADHD diagnosis, as manifested in the interplay of self-control, stigma and agency. In the concluding sections I use the empirical findings presented to propose a child–environment interaction model of ADHD diagnosis, and I demonstrate the importance of this model for UK policy.

The attention to ADHD diagnosis in this article complements two smaller studies that focused primarily on the implications of stimulant drug treatments for UK children (Singh, 2007, Singh et al., 2010). By putting children’s experiences in the analytic center, this body of work contributes to a process of, in effect, de-victimization of children diagnosed with ADHD, and allows for discovery of where children are truly vulnerable in relation to ADHD diagnosis, and where they are resilient.

## The VOICES study and research methods

The experiences of children described in this article were gathered as part of the VOICES study, an international project broadly interested in the social and ethical impacts of ADHD diagnosis and stimulant drug treatments for children. Between 2007–2010 the research team interviewed over 150 children ages 9–14 in the US and the UK. Children fell into one of three groups: children diagnosed with ADHD and treated with stimulants; children diagnosed with ADHD but not taking stimulants; and children without a psychiatric diagnosis.

Diagnosed children were recruited via university clinics in the US, and NHS Trusts in the UK; efforts were made in both countries to achieve geographic variety, within the limits imposed by time and budget considerations. Children without a psychiatric diagnosis were recruited using a variety of methods, including newspaper adverts, a market research company, posting flyers in schools and libraries, and word of mouth. We matched children broadly by age, gender and socio-economic status (measured using the Hollingshead 2-factor index of social position (Hollingshead, 1957)) within and across national cohorts, but we were unable to obtain matches for all the girls in the study, due to difficulties identifying and recruiting UK girls into the study.

We recruited children through clinics and physicians because we wanted to achieve a group of diagnosed children whose diagnoses were of a high standard – quite possibly higher than in a non-clinically recruited cohort. Still, the resulting group of diagnosed children represented behavioral difficulties across a spectrum of intensity and impairment, thus verifying many clinical researchers’ claims that ADHD is a heterogeneous and dimensional disorder. Ethics approvals were obtained in the UK through the NHS Clinical Research Ethics Committee and in the US through relevant university IRBs (Institutional Review Boards).

Methods and data analysis in the VOICES study were informed by Urie Bronfenbrenner’s model of the ecological niche (Bronfenbrenner, 1979). The model suggests that children’s behavioral development must be seen as a fundamentally situated and relational process in which there is an ongoing and mutual process of shaping and of transformation between child actors and their immediate and proximal social and physical spaces.

Data collection, analysis and management strategies aimed at collecting sufficient information on each child to enable building an accurate portrait of the child and his or her ecological niche. In addition to interviewing children, the research team spoke to many parents, clinicians and teachers. We visited parent support groups, schools and clinics, both to give talks and to learn from the people present. Notes of these discussions were taken, and questions and comments were sometimes followed up in the case of discussions with professionals. Information about local services and communities was gathered during these conversations, as well as by site visits, and visits to children’s homes and neighborhoods.

Parents filled out standardized questionnaires about their child’s behavior and diagnosis; and completed a demographic questionnaire. Children filled out the Harter’s Self-Perception Questionnaire at the end of the interview (Harter, 1985) which provided a quantitative account of a subset of the topics raised in the qualitative interviews.

Children took part in a one hour, semi-structured one-on-one interview, which was comprised of questions, a guided drawing task, a vignette, standardized pictures, a sorting task and sentence completions. All these elements were intended to prompt and guide conversation with children around different topics; the interview guide had been developed during a pilot study (Singh, 2007).

In all, four different female interviewers conducted the interviews in this study, thus diversifying the researcher-participant dynamics and contributing to the reliability of findings. Interviewers received training and were observed conducting at least two early interviews in order to ensure that they had a comprehensive understanding of the tasks and questions outlined in the semi-structured questionnaire. Interviews were digitally recorded and transcribed using a transcription service; standardized questionnaires were coded following manualized instructions and will not be discussed in this article. Each interview was read several times and then coded thematically by the primary investigator. Themes were further broken down into categories, and the relationships among categories were specified (Strauss & Corbin, 1990). An integrated approach (Bradley, Curry, & Devers, 2007) was used to develop both a ground-up coding frame, and a deductive ‘organizing framework’ for the kinds of codes used. A coding frame had been drawn up earlier, and discussed in a team of 3 other coders (none of whom had conducted interviews in the study) who each coded the same 6 transcripts independently. This process allowed for the verification and differentiation of codes through group discussion, and resulted in a coding frame that achieved high standards of agreement and transparency (Miles & Huberman, 1994). NVIVO and SPSS were used to manage the data streams.

## ADHD in the UK

By the end of the VOICES study, each folder on a child participant included the qualitative and quantitative data, notes on discussions with the child’s caregivers, any notes taken during or after the interview, and any relevant memos. For a subset of children we interviewed, this material was transformed into a case presentation, with an introduction crafted from the qualitative information we received, and a narrative of the child’s experience, using the child’s words as recorded at different points during the interview. To illustrate key characteristics of a dominant ecological niche we encountered in the UK, as well as common experiences of children within this niche, I present an abbreviated version of such a case below.

### Shaun

Shaun lives in a large village on the outskirts of a city in the middle of England, with his mother and father and two younger siblings. He is 12 years old, White, lower-middle class (Class III as measured by the Hollingshead index), and attends a state school. Shaun was diagnosed by a consultant psychiatrist with ADHD three years ago, and he has been taking extended release Concerta for the past 2 ½ years. Shaun’s mother gives him Omega-3 supplements and has experimented with dietary modifications to manage his symptomatic behaviors.

*ADHD is like behavior, just anger, like, temper; it’s like sometimes I feel really cross with other people and I just want to go lashing, lashing out. I’ll like kick or punch kids. [Other kids at school] know they can wind me up easily so they do it again and again and I can’t walk away that easy. [My dad says] not to throw the first punch, but if I get punched, I have to fight back. Teachers are not effective. They don’t help. They’re always shouting, but no one listens to them…about half of them forget that you’ve actually got ADHD when you’re in their classroom. My mates look out for me. If I’m running toward somebody they would either tackle me or hold me down or something. That’s what good mates do for each other. They know what I’m like. I’ll see them after school sometimes. Depends on whether I think I should do my homework… [My friends and I] play X-box and sometimes we’ll get out to a field to play footy.*

To understand Shaun in the context of the ecological niche model, it is important not to focus solely on his demographic characteristics, eg. ethnicity, social class and gender. Shaun represents a dominant ecological niche rather than a dominant type of individual child, and individual demographic variables do not define the niche. Family, neighborhood and broader social dynamics are equally important to understanding an ecological niche.

#### Family and community

From the perspective of the children we interviewed, even larger village neighborhoods like Shaun’s are still rather intimate geographic and social settings. Children are more likely to be allowed to walk or bike the streets in order to get from one place to another; and they have access to local parks, high streets and playgrounds to hang out. We often hear about visits with grandparents and other relatives, or that relatives regularly help with childcare. Reports on family and community life from children in our study find support in larger studies, which document a continuing active exchange of support within the extended UK family and suggest that ‘the character of family life and of the relationships’ within UK communities has remained largely unchanged in 40 years (Charles, Davies, & Harris, 2008, xii).

Such intimacy and support can be beneficial for a child’s feeling of safety and belonging in a community. However, it also reflects a problem of low social mobility within UK society (Aldridge, 2003); and correspondingly low aspirations for social mobility among some young people (Nunn, Johnson, Monro, Bickerstaffe, & Kelsey, 2007). University attendance – which often generates both geographic and social mobility – is not considered the natural next step after secondary school if parents do not have a network of university-educated peers (Morris & Rutt, 2005). Shaun’s mother attended the same high school that he now attends; and his father attended high school in the next town. Neither of Shaun’s parents attended university. Shaun is also unlikely to attend university, although he will probably go on several courses in order to receive certifications necessary for skilled work and he may one day own his own business, like his father does.

#### Schools

The relative lack of academic aspiration, combined with the relative lack of social movement within Shaun’s ecological niche, means that longstanding social patterns are more difficult to shift, even if they are not encouraging individual and social flourishing. Schools, as repositories for community values, can have embedded negative social patterns (Sutton, Smith, Deardon, & Middleton, 2007). At least this is one way of understanding the pervasive and apparently pernicious presence of a state school-based culture of aggression we hear about from children like Shaun:

*I got in a fight with someone and gave him a nosebleed because… I was in PE and I think someone was taking the mickey out of a kid [making fun of him] and I thought it was me… so I decided to punch him…at my last school that happened most days*. Lionel, age 12

Parents who struggled through the same schooling system tell us that they feel little agency in tackling these negative social patterns; but they do instruct their children on the rules of engagement:

*My dad was like my age, he got in a fight and got kicked in his privates so bad that he needed an operation… Last week this boy did the same to me but not as bad… My dad says don’t start a fight but fight back if you get hit*. Aaron, age 12

Bullying is widely reported to us by children in UK state schools. In our interviews bullying includes fighting, name-calling, pushing, shoving, stealing and ridiculing; it takes place in person or on the internet (cyber-bullying). About three-quarters of the diagnosed children we meet have been involved in physical fights at school, either as aggressor or as victim, or in both roles. Being a girl does not protect against bullying involving physical aggression; indeed, several of the diagnosed girls we meet in the UK have been severely bullied but are also aggressive.

*[This boy] he keeps pushing me, pushing me to be, make me angry. And he hits me, he bribes me, he takes money from me. He does anything to annoy me. He knows I’m easy to wind up and he knows I’m easy to get my anger up. I just start hitting him like he hits me*. Charlotte, age 11

The experiences of children in the VOICES study intersect with national trends. Bullying has been a key UK national health and education policy agenda item for at least the past decade, but there are no official government statistics on the problem and national survey data is sparse, contentious, and limited in scope. The Tackling Bullying Report, which in 2003 surveyed just over 1000 students, found that 51% of Year 5 students (ages 9–10) and 28% of Year 8 students (ages 12–13) experience bullying during a school term. A substantial proportion of students in both age groups report bullying involving physical aggression, including pushing, hitting and kicking. Among Year 8 children, bullying is less frequent, but experiences of physical aggression as part of bullying are more common. The survey found no significant differences between male and female students in terms of frequency of bullying, although it found that reports of psychological bullying were higher among girls (Oliver & Candappa, 2003).

A further peculiar and troubling feature of the culture of aggression in UK state schools is that it extends into the classroom, where it can involve teachers. An occasionally harsh, disengaged teaching style in state schools is targeted in recent policies that aim to transform UK schools into a less conflict-oriented, less punitive culture (Layard & Dunn, 2009). At the same time, the UK government has recently sanctioned the use of force on disruptive pupils by teachers, in an effort to provide teachers with more tools to manage what are viewed as unacceptably high levels of ‘violence’ in state schools (http://news.bbc.co.uk/1/hi/education/6519455.stm). In the course of our interviews, children without diagnoses of ADHD frequently comment negatively on the extent to which loud arguments between teachers and ‘naughty’ or ‘rude’ children take up teachers’ energies:

*There’s kids that just do really rude stuff, like arguing with teachers*… *they’ll just do it for fun, or to get people wound up, like attention seekers*… *There’s a lot of shouting*. Luka, age 13

*I wish people would notice that I’m, like, behaving well, but most people concentrate on bad behavior. Like teachers*…*they are too busy sorting out the naughty kids*. Salma, age 10

For children with ADHD diagnoses, shouted arguments with teachers are an everyday experience:

*It’s normal for kids to get upset and shout at teachers*. Nicholas, age 14

*My teacher shouts quite a lot. It’s a bit scary*. Pablo, age 11

*I get stood up in front of the class and well, stand there, and he start shouting at me*. Jared, age 11

In such classrooms, ADHD behaviors become part of an ongoing struggle between students and teachers over appropriate behaviors, with students alleging that teachers behave in ways that are disrespectful, aggressive, and out of control, leaving them little incentive to manage their own behaviors. Indeed, the intense focus on negative behaviors in UK state school classrooms may mean that behavior, not learning or academic performance, becomes children’s primary concern. Children like Salma, who are well behaved, feel ignored. Diagnosed children feel overwhelmed with loud, aggressive negative attention; they too long for praise for good behavior. As Shaun puts it, good academic performance under these circumstances is not a priority; it’s ‘a bonus’:

*I feel good about my behavior if I’d been, like, good all that time and if someone’s like rewarded me at school, like and said how good I am, or they write on their own to my parents saying how good I’ve been, something like that would make my day… It’s just like normal praise really. Like if I do good on tests and everything that is a bonus, but if I’ve been good because I’ve not been, like, disrupting or anything all day, because I’ve controlled my ADHD, that’s like, even better.*

Like many diagnosed UK children we interviewed, Shaun even responds to a question about his future aspirations in terms of behavior:

*In the future I guess I want to be less naughty*.

UK children with ADHD diagnoses, who share the key characteristics of Shaun’s ecological niche, worry less about *doing* well and more about *behaving* well. As a point of comparison, doing well is a primary preoccupation in a dominant ecological niche represented in our US sample. This is a performance-focused niche in which children infrequently experience physical or verbal aggression from school peers or teachers, due in part to institutional prohibitions on such behaviors. These US children tend to view ADHD in terms of academic performance, associate good behavior with good grades, and they generally answer future-oriented questions in terms of their professional aspirations:

I*n the future I, like, umm, I want to be a chef*. Graham, age 11

While it is possible to associate academic aspirations and levels of aggressive behavior in schools with social class, the overall niche ‘ethos’ seems more important to children’s experiences of ADHD behaviors, and to the expression of these behaviors, than any single demographic variable. For example, in a different US ecological niche, where economic and familial resources are more scarce and there are limited supports in place for young people, children report higher levels of aggressive behavior and bullying among peers and in schools. Children in these niches are more likely to experience their ADHD behaviors in the context of aggressive altercations. Still, in our limited sample, children from such US niches also reference the importance of school performance and future aspirations in relation to their ADHD behaviors.

Similarly, UK students inhabiting well resourced, highly educated niches are more likely to mention the importance of school performance when discussing their ADHD behaviors. However, the small number of students we interviewed from such niches also report regular incidents of bullying and some participation in aggressive altercations at school. These students interpret their ADHD behaviors in light of these experiences, as well as in relation to academic performance expectations.

## ‘Wound up’: A disorder of anger and aggression

A cultural pattern of bullying and aggression in UK state schools (Cawson, Wattam, Brooker, & Kelly, 2000) provides a social channel for expression of a child’s difficulties with self-control, such that ADHD is widely known by its colloquial definition: ‘anger.’ Among UK school children, ‘anger’ in relation to ADHD does not refer to an experience of feeling outraged in response to a real or imagined injustice. Instead, ‘anger’ refers to a struggle with self-control in aggressive situations. A poorly controlled short fuse is probably the most common understanding of ADHD that we encountered in the UK; and children who exhibit apparently uncontrolled aggressive behaviors will be suspected of having ADHD by other children.

*I think I know one person in my school who has ADHD. He’s a bit mad, like, he gets really angry…. He like, if somebody teases him…he like, he can’t get it out of his head… He jumps on people’s backs and squeezes the backs of their necks*. Olli, no diagnosis, age 10

Children with ADHD diagnoses also experience their disorder in terms of poor self-control in response to taunts:

*At birthday parties I think, ‘well I’m a normal person like everyone else’… But then when someone’s being horrible to me that’s when I know that I’ve got it… because I get wound up so easily. My heart starts to beat faster and like I go red and clench my fists*… Charlotte, age 11

It is significant that ADHD is associated with a lack of *emotional* self-control in the UK. ‘Anger’ is, after all, not a behavior. This lack of control over emotions is arguably the most stigmatizing dimension of UK ADHD, in part because emotional self-control is highly valued in the UK. Children’s implicit understanding of the value of self-control is illustrated by a common, and unique (as compared to US children) experience among UK children with ADHD diagnoses: Other children go out of their way to ‘wind them up.’ The game is to get children known for having a short fuse to lose control and start fighting:

*Because I told [peers] about my ADHD, they thought if they could wind me up I’d get really upset and they love to do that. I thought if I just ignored it then they would get really bored of doing it and stop… But they didn’t… [Teachers don’t help]. They just say ignore it…[They] know what I’m like but I don’t think they really know how hard it is for me to cope*. Heidi, age 11

While bullying involving some level of physical aggression is common in UK schools, children with a diagnosis of ADHD are especially likely to be drawn into aggressive altercations, more often as victims but also as victimizers. Not only are they more likely to be drawn into these situations, they are also more likely to experience a distressingly rapid escalation of anger and loss of self-control in the process. It is this loss of self-control – rather than aggressive behavior per say – that marks diagnosed children, both to others, and, as Charlotte says above, to themselves.^1^

This interplay of individual behavioral dispositions and environmental factors may also explain why ADHD symptoms in the UK are such an intensely – and again uniquely, as compared to most US children – somatic experience. UK children are often quite literally engaged in fight or flight scenarios:

*My brain beats really fast and that’s why I mess around and get in fights… I get really bad headaches when it’s happening. It’s not meant to go that fast*. Simon, age 9

*My body starts feeling really rough and hot… it feels sort of really aggressive*. Laurence, age 10

*When I’m angry my body feels like it’s going to explode… I just go around hitting anything I see*. James, age 11

Among UK children occupying a dominant ecological niche, ADHD is not just a cognitive problem of focus and attention, it is a significantly impairing, highly somatized difficulty with self-control in the context of an actively hostile environment. But despite the stigmatizing association of ADHD diagnosis with uncontrolled anger and aggression, diagnosed children should not be seen as victims of their environment – or of their poor capacity for self-control. In the next section I turn to an investigation of what children do with an ADHD diagnosis – specifically, how diagnosed children negotiate moral agency in the complex terrain of the UK school playground.

## ADHD on the playground: engaging and avoiding fights

### Self-control and moral obligations

UK children learn most of what they understand about their ADHD diagnosis on the school playground. Here children are left largely to their own devices when encountering and resolving conflicts; children tell us that there is little direct supervision by teachers while they are outside, and teachers who are there appear powerless or unwilling to interfere with bullying and aggression:

*I’ll go and tell a teacher but the teacher says, ‘You keep telling us and it’s getting annoying.’ So there’s nothing really very much we can do. So it really just leaves us to sort it out. And then it just sorts it out over a fight*. Ned, age 12

In the absence of strong normative limits on interpersonal behavior, children depend on friendships to resolve playground conflicts. Because of their difficulty with self-control, children with ADHD rely particularly on their friends to stand up for them in a fight, or to talk them down from one:

*[My friends will] jump on me and hold my arms back and just restrain me, a bit. And in the end I just end up just calming down… but sometimes I can’t and I break away*. Pat, age 11

The frequency with which UK children reference the importance of friendships in helping them manage their behavior suggests the extent to which behavioral self-control, in reality, is rarely a matter of autonomous decision-making on UK school playgrounds. Sometimes fights can be prevented. Sometimes they cannot be. It depends on medication. It depends on mood. But it depends most heavily on friendships; and on what the bullies say.

Sometimes what the bullies say means that it’s not possible to avoid fighting. If the bullies say, *You’re a twat and your mum’s got a big fat ass*; or if they say, *What’s wrong little boy? Are you upset that your mum is dead?* then a boy must fight; he has no choice. Girls do not seem beholden to this particular rule of engagement. For boys, however, mothers are off limits; and invoking her triggers a moral obligation to fight. This is true even if boys do not have good relationships with their mothers: *I don’t let anyone curse her off; only I’m allowed to*. All boys know this, so if winding up a boy with ‘anger problems’ is not working, this will be the final resort. Even boys who know they would end up on the worst end of a fight, or those who hate fighting, will fight under these circumstances, and hope that their friends will come to their defense.

### Mobilizing ADHD diagnosis

Children with ADHD diagnoses do not just rely on their mates to come to their assistance in conflict situations. They also actively protect their friends. Although an ADHD diagnosis can invite bullying, we frequently hear that children use ADHD diagnosis to help avoid fights and protect friends:

*If they’re really bothering me, or bothering one of my mates, I’ll just go into my ADHD. I’ll flip on them and get really scary… They know not to mess with me; they know I’ll go mental and really hurt someone*. Lionel, age 11

When a child ‘goes into his ADHD’ he consciously inhabits the label and mobilizes the behavioral and the social resources of the diagnosis. Lionel himself exploits the stigma of ADHD, and he draws upon his somatic capacity to ‘go mental’ and ‘to flip.’ He hopes the result will be that he and his friends will be left alone.

Such conscious habitation of the ADHD label by children tells us that tools such as ADHD diagnosis, developed to help manage and make sense of unruly and unproductive biology, are not hegemonic over identity or, indeed, over somatic experience. In fact I would argue that in the UK, children with ADHD diagnoses currently come to know and to control their behavior not primarily through the language, or even the tools, of psychiatry, neuroscience and genetics, but through the moral obligations conferred upon them by the bonds of friendship and loyalty. Those bonds are a primary motivation, and a primary vehicle, for modulating their capacity for self-control:

*My mates look out for me. If I’m running toward somebody they would either tackle me or hold me down or something. That’s what good mates do for each other. They know what I’m like*. Shaun, age 12

Under this ‘relational regime,’ the reflexive space is open to moral decision-making that is motivated by care for self *and* others, and by relationally-informed ideals of justice. In this space, children with ADHD can mobilize self-control creatively to resolve playground conflicts without aggression, and to protect friends:

*I had a mate who was always getting picked on, and you know, he wasn’t really strong enough to fight so I would like go in there and use my ADHD to get them to leave him alone*. Aaron, age 12

Mobilizing ADHD in this way is prosocial; it fosters bonds between children who then ‘have each other’s backs’; they watch out for each other. Indeed, Aaron reminds us that excusing an individual child for aggressive behaviors on the basis of reduced capacity for self-control elides the social and relational processes that both incite and help to prevent that child from fighting. Self-control is indeed a finely tuned interaction between individual and environmental factors; and among UK school children, friendships help children make a mental space for moral deliberation, discover their moral and behavioral boundaries, and establish a framework for social justice.

### Exploiting ADHD diagnosis

Playing up the stigma of ADHD diagnosis is a double-edged sword: when used for prosocial ends it is a positive form of agency; when used for selfish ends, it ultimately diminishes agency. UK children report exploiting their ADHD diagnosis, primarily as an excuse for bad behavior:

*I don’t get punished for nothing. It’s easy to get away after fights because I have ADHD. I just make puppy eyes and it gets me round everything with my teachers*. Alan, age 10

Unlike US children, who rarely admit to using ADHD as an excuse for their behavior (because they believe it is wrong, but also because niche dynamics strongly encourage them to keep their diagnosis a secret), almost all UK children say they have used ADHD as an excuse. Frequently, it works, at least to a degree. From children’s perspectives, many teachers and headmasters seem to have categorical views about ADHD: either they believe the disorder is not real and they make no allowances for a child with ADHD; or they believe it is real and excuse diagnosed children’s aggressive behaviors (but fail to enact effective educational supports for the child). Children report that school personnel tell other children to stay away from them because they have ADHD, and they give lesser punishments to students with ADHD diagnoses.

Children have ambivalent feelings about the practice of ADHD exploitation: they are glad for the opportunity to get out of punishments, but they know this practice encourages the narrow, stigmatizing conflation of ADHD, anger, and aggression: *The headmaster just thinks ADHD means we’re violent.* These ambivalent feelings are one reason why some children do not want to tell their friends about their disorder. Laurence explains:

*I’m afraid that if I tell my friends about ADHD they’ll use it as an excuse to like, help me get off after fights or something. But maybe I could control how I behaved*. Laurence, age 10

As Laurence suggests, the more ADHD is socially available as an excuse for behaviors, the less control a child with ADHD has over how he is seen, and indeed, how he sees himself. Even well-meaning friends threaten a child’s capacity for self-determination when they use ADHD as an excuse on his behalf. Exploiting ADHD fuels the short fuse stereotype, and may encourage a rather remarkable social phenomenon we encountered several times at particularly ‘hard’ schools -- ADHD-adoption:

*Yeah, I have like told people I got ADHD cos it makes them leave you alone. They’re nervous that you might really hurt them if you get wound up*. Paul, age 13, no diagnosis

ADHD-adoption is when undiagnosed children spread the word that they have ADHD in order to build up their personal armament against harassment, thereby further instantiating the stereotype. In the process, ‘real’ ADHD is increasingly diluted in meaning and in value, while the short fuse stereotype is empowered.

Children with ADHD diagnoses complain that …*kids will just pretend that they have [ADHD] to get out of stuff.* Such pretense also means that teachers’ suspicions about the validity of ADHD diagnoses have traction, given that ADHD is actually being used as an excuse by children who do not have a diagnosis. The practices of ADHD-adoption and ADHD exploitation translate into a perpetual discourse of suspicion in some UK schools, about who might have ADHD, whether diagnosed or undiagnosed, and who might be pretending to have it. It is difficult for children to know the true diagnostic status of other children in the school, even though many children are forthcoming about their diagnoses with other children. As Freddy, age 11, says: *My friend says he has it [ADHD] too but I’m not really sure that he’s telling the truth. Maybe he just thinks he has it*.

## What is ADHD?

Given the controversy over the validity of the ADHD diagnosis, it is tempting to argue that this presentation of what ADHD means in the UK, and children’s exploitation and mobilization of those meanings, suggests that ADHD diagnosis represents not a ‘real’ disorder but rather a de-valued set of behaviors. For example, one might say that in a context where self-control in response to aggressive situations is highly valued, a lack of self-control is more likely to be interpreted as a disorder – just as in contexts where school success is highly valued, poor school performance is more likely to be interpreted as a disorder (a common critique of ADHD diagnosis in the US).

A further objection to the validity of ADHD diagnosis based on the data presented in this article, could be that in a different context, the aggressive behaviors of UK children would be associated not with ADHD but with Conduct Disorder (in the US, for example), or with heroism (in ancient Greece, for example). This too suggests that ADHD does not refer to a stable, universal disorder; rather it is a convenient catch-all category.

I take it as a given that behavioral interpretation is to some extent culturally relative and that diagnostic practices index social values. But this does not necessarily invalidate ADHD diagnosis; it does mean that diagnostic practices should pay close attention to the environment and acknowledge, in a systematic and reflexive way, the substantial traces of context and culture that behavioral interpretation, and behavior itself, carry. To assist this, sociological models of diagnosis should move beyond reductive arguments that locate disorder either in the child or in the environment, towards more complex models that allow for the interplay between the two, and view diagnosis as part of that interplay, not separate from it.

The discussion in this article suggests the following model: A child’s difficulty with behavioral self-control finds its expression in, is shaped by, and gives shape to, a normative behavioral channel. In the UK state school environment, one of these channels is aggression, which can intensify symptomatic behaviors and can inspire behaviors in diagnosed children that inflame the environment. Clearly environment plays a critical role in shaping children’s behavioral capacities – and children’s behaviors in turn shape the environment. In a modal US school environment, where peer aggression is low but pressure to perform well in school is higher, we found that difficulties with self-control were more likely to be expressed in the context of a ‘performance’ channel.

Identifying these channels in the model illuminates the embodied nature of symptomatic behaviors themselves and suggests why a difficulty with self-control can look behaviorally (and feel) quite different across different contexts. For example, recent research in the US suggests the presence of another channel through which a difficulty with behavioral self-control can be expressed: food. In a US population study, ADHD symptoms were associated with an increased risk of obesity (Fuemmeler et al, 2010). Presumably further sociological investigation of the ‘food channel’ would highlight the role of social and cultural factors in the work this channel does to engage behavioral dispositions and environmental factors and to help shape a distinctive phenotype.

Even this primitive articulation of the ‘channel model’ motivates more comprehensive thinking about intervention. A diagnosis of psychiatric disorder is meant to predict course and outcome, and to suggest the best treatment. If ADHD diagnosis refers to patterned *relationships* between behavioral dispositions and environmental factors, then it is possible to ask where early interventions and treatment should focus. What needs adjusting in order to ensure good outcomes? A child-focused model of ADHD often closes off environmental avenues of intervention and implies a causal logic that propels a series of psychotropic drug treatments. Children tell us that drug treatments work, at least in the earlier stages of treatment. But treatment adherence and effectiveness would surely be amplified if physicians were given more resources and incentives to also facilitate relevant environmental adjustments by working, in the UK example, with families and schools.

## Conclusion: policy matters

Moving to a sociologically informed model of ADHD is important in the UK. Recently, a conceptually coordinated series of UK policy reports has framed ADHD specifically as a deficit in ‘self-regulation skills’ and as a threat to national prosperity:

*[ADHD] produces an estimated lifetime earnings cost of £43,000, suggesting that substantial benefits would accrue to the individual (and to the economy) from interventions that would reduce these problems*. (Foresight Report on Mental Capital & Wellbeing, 2009:101)

Painting a picture of a generation of alienated, unmotivated and anti-social youth, these UK policy reports focus on ‘character’ building as a necessary component of interventions. Character development is seen as part of the science of cognitive development, and not a moral issue: ‘*character represents a set of life skills rather than a moral disposition’*. (Lexmond & Reeves:2009:12)

As this article has shown, for many UK children self-regulation is not only a cognitive skill but also a moral behavior; and a child’s cognitive and behavioral capabilities – and thereby his moral potential - are intimately linked to relational obligations. Indeed, these obligations can motivate behavioral self-control: children report making a priori decisions to fight when morally obliged, and they report mobilizing their ADHD to avoid fighting in conflict situations. It is important that policy interventions capitalize on the available social mechanisms that potentiate children’s ‘character capabilities’. This route of capitalization is more likely to inspire behavioral change within communities by encouraging children to build on their capacity for agency, and their social and moral wisdom. At the same time, policy-makers should work to dismantle the school-based culture of aggression that is arguably a generative, and surely a sustaining ground for UK-style ADHD: a disorder of anger and aggression.

The symptoms of ADHD do not emerge, grow and take shape solely, or even predominantly, as a consequence of primary biological deficits. The tools of social science in general, and of sociology in particular, can help to develop complex models of psychiatric diagnosis that deepen scientific and public understanding and promote relevant, dynamic and effective interventions.

## Footnotes

1 In my experience, UK clinicians readily recognize how a school-based culture of aggression complicates understanding of children diagnosed with ADHD. The ‘naughty child’ stereotype is widely seen to undermine the validity of ADHD diagnosis in the UK.

## References

[bib1] Aldridge S. (2003). The facts about social mobility: a survey of recent evidence on social mobility and its causes. New Economy.

[bib2] Barkley R. (1997). ADHD and the nature of self-control.

[bib3] Bradley E.H., Curry L.A., Devers K.J. (2007). Qualitative data analysis for health services research: developing taxonomy, themes, and theory. Health Research and Educational Trust.

[bib4] Bronfenbrenner U. (1979). The ecology of human development.

[bib5] Butler J. (2005). Giving an account of oneself.

[bib6] Cawson P., Wattam C., Brooker S., Kelly G. (2000). Child maltreatment in the United Kingdom: A study of the prevalence of abuse and neglect. https://www.nspcc.org.uk/Inform/publications/Downloads/childmaltreatmentintheUKexecsummary_wdf48006.pdf.

[bib7] Charles N., Davies C.A., Harris C. (2008). Families in transition: Social change, family formation and kin relationship.

[bib9] Conrad P. (1976). Identifying hyperactive children: The medicalization of deviant behaviour.

[bib10] Foresight Mental Capital and Wellbeing Project (2008). Final project report.

[bib11] Foucault M. (1975). Discipline and punish.

[bib12] Fuemmeler B.F., Østbye T., Yang C., McClernon F.J., Kollins S.H. (2010). Association between attention-deficit/hyperactivity disorder symptoms and obesity and hypertension in early adulthood: a population-based study. International Journal of Obesity.

[bib13] Fukuyama F. (2002). Our posthuman future.

[bib14] Goffman E. (1961). Asylums.

[bib15] Graham L. (2008). From ABCs to ADHD: the role of schooling in the construction of behaviour disorder and production of disorderly objects. International Journal of Inclusive Education.

[bib16] Hacking I. (1999). The social construction of what?.

[bib17] Haimes E. (2002). What can the social sciences contribute to the study of ethics?: theoretical, empirical and substantive considerations. Bioethics.

[bib18] Harter S. (1985). Manual for the self-perception profile for children.

[bib19] Healy D. (2002). The creation of psychopharmacology.

[bib20] Hollingshead A.B., Miller D.C. (1957). Hollingshead two factor index of social position. Handbook of research design and social measurement.

[bib21] Horwitz A. (2002). Creating mental illness.

[bib22] Johannesson I. (2006). Strong, independent, able to learn more…: Inclusion and the construction of school students in Iceland as diagnosable subjects. Discourse: Studies in the Cultural Politics of Education.

[bib47] Kirk, S., & Kutchins, K. (2003). *Making us crazy*. New York: Free Press.

[bib23] Layard R., Dunn J. (2009). A good childhood: Searching for values in a competitive age.

[bib24] Lexmond J., Reeves R. (2009). Building character.

[bib25] Lindemann H.N. (2010). Speaking truth to power. HCR.

[bib26] Lock M. (2001). The tempering of medical anthropology: troubling natural categories. Medical Anthropology Quarterly.

[bib27] Maturo A. (2009). Medicalization, multiplication of diseases and human enhancement: round table with Kristin Barker, Ivo Quaranta, Martijntje Smits and Louis Francois Vedelago. Salute e Societa.

[bib48] Morris M., Rutt S. (2005). Evaluation of Aimhigher: Excellence challenge. Aspirations to higher education: One year on. http://https://www.education.gov.uk/publications/eOrderingDownload/RR651.pdf.

[bib28] Miles M.B., Huberman M. (1994). Qualitative data analysis: A sourcebook of new methods.

[bib30] Nunn A., Johnson S., Monro S., Bickerstaffe T., Kelsey S. (2007). Factors influencing social mobility. http://eprints.hud.ac.uk/6057/.

[bib31] Oliver C., Candappa M. (2003). Tackling bullying: Listening to the views of young people. http://www.dfes.gov.uk/research/.

[bib32] Polanczyk G., de Lima M.S., Horta B.L., Biederman J., Rohde L.A. (2007). The worldwide prevalence of ADHD: a systematic review and metaregression analysis. The American Journal of Psychiatry.

[bib33] President’s Council on Bioethics (2003). Beyond Therapy: Biotechnology and the pursuit of happiness.

[bib34] Rose N. (2007). The politics of life itself: Biomedicine, power and subjectivity in the twenty-first century.

[bib35] Scheff T.J. (1974). The labelling theory of mental illness. American Sociological Review.

[bib36] Scheffler R.M., Hinshaw S.P., Modrek S., Levine P. (2007). The global market for ADHD medications. Health Affairs.

[bib37] Singh I. (2005). Will the ’real boy’ please behave: Dosing dilemmas for parents of boys with ADHD. The American Journal of Bioethics.

[bib38] Singh I. (2007). Clinical implications of ethical concepts: the case of children taking stimulants for ADHD. Clinical Child Psychology and Psychiatry.

[bib39] Singh I. (2008). Beyond polemics: science and ethics of ADHD. Nature Rev Neuroscience.

[bib40] Singh I., Kendall T., Taylor C., Mears A., Hollis C., Batty M., Keenan S. (2010). Young people’s experience of ADHD and stimulant medication: a qualitative study for the NICE guideline. Child and Adolescent Mental Health.

[bib41] Sonuga-Barke E.J. (2005). Causal models of attention deficit/hyperactivity disorder: from common simple deficits to multiple developmental pathways. Biological Psychiatry.

[bib42] Strauss A.L., Corbin J. (1990). Basics of qualitative research: Grounded theory procedures and techniques.

[bib43] Sutton L., Smith N., Deardon C., Middleton S. (2007). A child’s-eye view of social difference.

[bib44] Timimi S. (2009). The commercialization of children’s mental health in the era of globalization. International Journal of Mental Health.

[bib45] Vega Balbas R. (2007). Bioidentity and medicalization: an insight into ADHD from biopolitics. Arteterapia.

[bib46] Walker M.U., Lindemann H., Verkerk M., Walker M.U. (2009). Introduction: Groningen Naturalism in bioethics. Naturalized bioethics.

